# Cellulolytic potential of thermophilic species from four fungal orders

**DOI:** 10.1186/2191-0855-3-47

**Published:** 2013-08-19

**Authors:** Peter Kamp Busk, Lene Lange

**Affiliations:** 1 Department of Biotechnology Chemistry and Environmental Engineering, Aalborg University, A.C. Meyers Vænge 15, 2450 Copenhagen, SV, Denmark

**Keywords:** Thermophilic fungi, Endoglucanase, Cellobiohydrolase, Cellulolytic potential

## Abstract

Elucidation of fungal biomass degradation is important for understanding the turnover of biological materials in nature and has important implications for industrial biomass conversion. In recent years there has been an increasing interest in elucidating the biological role of thermophilic fungi and in characterization of their industrially useful enzymes. In the present study we investigated the cellulolytic potential of 16 thermophilic fungi from the three ascomycete orders *Sordariales, Eurotiales and Onygenales* and from the zygomycete order *Mucorales* thus covering all fungal orders that include thermophiles. Thermophilic fungi are the only described eukaryotes that can grow at temperatures above 45°C. All 16 fungi were able to grow on crystalline cellulose but their secreted enzymes showed widely different cellulolytic activities, pH optima and thermostabilities. Interestingly, in contrast to previous reports, we found that some fungi such as *Melanocarpus albomyces* readily grew on crystalline cellulose and produced cellulases. These results indicate that there are large differences in the cellulolytic potential of different isolates of the same species. Furthermore, all the selected species were able to degrade cellulose but the differences in cellulolytic potential and thermostability of the secretome did not correlate to the taxonomic position. PCR amplification and sequencing of 22 cellulase genes from the fungi showed that the level of thermostability of the cellulose-degrading activity could not be inferred from the phylogenetic relationship of the cellulases.

## Introduction

Fungi are important organisms for degradation of plant material in nature. They achieve this by means of secreted enzymes that are stable even under harsh environmental conditions. These same properties make the fungal enzymes suitable for industrial use. One example is fungal cellulases that are deployed in biorefineries for conversion of biomass to fermentable sugars and in the paper, textile and detergent industries (Karmakar and Ray 2011; Kuhad et al. 2011).

The cellulases are classified in the glycoside hydrolase (GH) families (http://www.cazy.org) (Henrissat and Davies 1997). Several different strategies involving many enzyme classes are used in natural degradation of recalcitrant biomass (Dashtban et al. 2009). However, industrial use of cellulases has mainly been focused on endo-1,4-β-D-glucanase and two types of cellobiohydrolases acting respectively from the reducing and from the non-reducing end of the polymer (Banerjee et al 2010). Additional industrially used cellulose-degrading enzymes are β-glucosidases, which degrade β-D-glucose oligomers to glucose and the GH61 proteins, which boost cellulose decomposition by oxidative degradation of the glucose polymer (Harris et al. 2010; Langston et al. 2011; Quinlan et al. 2011; Westereng et al. 2011).

Biomass decomposition by mesophilic fungi has been extensively studied (Dashtban et al. 2009). Whereas enzymes from mesophilic fungi are typically effective at 50°C several thermophilic fungi produce more thermostable enzymes that can be used at temperatures up to 70°C (Murray et al. 2004; Parry et al. 2002; Venturi et al. 2002; Voutilainen et al. 2008; Wojtczak et al. 1987). This high temperature stability is an important asset for industrial use. For example, it has been shown that a mixture of thermostable cellulases exhibits high lignocellulose degrading capacity with a temperature optimum of 65°C (Viikari et al. 2007).

The known thermophilic fungi are either ascomycetes belonging to the orders *Sordariales*, *Eurotiales*, and *Onygenales* or zygomycetes of the order *Mucorales* (Berka et al. 2011; Morgenstern et al. 2012). These organisms are the only described eukaryotes that can grow at temperatures above 45°C. In nature, thermophilic fungi are typically found in compost, wood chip piles, stored grains, animal dung and other environments that are self-heating due to degradation of plant materials (Johri et al. 1999). Although such material often contains high concentrations of cellulose some thermophilic fungi are poor cellulose-degraders and seem to utilize sugars released by cellulolytic species in the biotope (Maheshwari et al. 2000). Therefore, thermophilic fungi can differ greatly in their cellulolytic potential.

The thermophilic fungus *Thermoascus aurantiacus* of order *Eurotiales* has been studied intensively. It grows readily on cellulose (Romanelli et al. 1975) and produces thermostable cellulases and other enzymes (Gomes et al. 2000; Hong et al. 2003; Hong et al. 2007; Khandke et al. 1989; Parry et al. 2001; Parry et al. 2002). One of the interesting enzymes produced by *T. aurantiacus* is a copper-dependent monooxygenase of the GH61 family (Harris et al. 2010). Several GH61 proteins degrade cellulose by an oxidative mechanism thereby boosting the action of cellulases (Langston et al. 2011; Quinlan et al. 2011; Westereng et al. 2011). The GH61 from *T. aurantiacus* exhibits high activity in a boosting assay (Harris et al. 2010). The high cellulolytic potential of this fungus is underpinned by the report that extracellular enzymes from *T. aurantiacus* release the same amount of sugars from pretreated switchgrass as the commercial cellulase blend Cellic Ctec2 (Novozymes, Bagsvaerd, Denmark) at the same protein load (McClendon et al. 2012). In addition to the high activity, the *T. aurantiacus* enzymes have higher thermostability than Cellic Ctec2, which probably consists mostly of enzymes from mesophilic fungi although the precise composition has not been disclosed by the manufacturer.

Other thermophilic fungi that produce thermostable cellulases are *Talaromyces emersonii* (Murray et al. 2004; Voutilainen et al. 2010), *Myceliophthora thermophila* (Roy et al. 1990), *Chaetomium thermophilum* and *Acremonium thermophilum* (Voutilainen et al. 2008).

In the present study we investigated the cellulolytic potential of isolates of 16 thermophilic fungi. The fungi were selected from the three ascomycete orders *Sordariales, Eurotiales and Onygenales* and from zygomycete order *Mucorales* thus covering all orders which harbor thermophilic fungal species.

All 16 fungi were able to grow on crystalline cellulose but their extracellular enzymes showed widely different cellulolytic activities, pH optima and thermostabilities. Furthermore, we used PCR with degenerate primers to amplify and sequence gene fragments of 22 new cellulases from these fungi. The phylogenetic relationship of the enzymes showed a better correlation to the fungal order than to the thermostability of the fungal cellulose-degrading activity.

## Materials and methods

### Fungi and growth conditions

Fungi were purchased from Centraalbureau voor Schim-melcultures, Utrecht, The Netherlands (Table 1). Unless otherwise indicated, growth experiments on different carbon sources were done essentially as described (Herr 1979). Briefly, the fungi were grown on minimal medium with 1% glucose for 3 days. Next, 500 μl of this culture was transferred to 5 ml of basal medium supplemented with the indicated carbon source with or without 0.5% yeast extract. The cultures were incubated with shaking (250 rpm) for the time indicated. The growth temperature was 45°C except for *T. emersonii* and *Scytalidium thermophilum* that were incubated at 37°C.

**Table 1:** List of fungi and growth on cellulose

**Order**	**Name**	**CBS no.**^**a**^	**Isolated from**	**Growth on Avicel**	**Abbreviation used in figs.**
*Sordariales*	*Chaetomium senegalense*	728.84	Plant remains	Poor^b^	Chsene
*Sordariales*	*Chaetomium thermophilum*	180.67	Typha, incubated strawand leaf mold	Yes	Chther
*Sordariales*	*Corynascus thermophilus*	406.69	Mushroom compost	Yes	Cother
*Sordariales*	*Melanocarpus albomyces*	638.94	Chicken nest straw	Yes	Malbo
*Sordariales*	*Remersonia thermophila*	540.69	Mushroom compost During peak heating	Yes	Rether
*Sordariales*	*Scytalidium indonesiacum*	259.81	Soil	Yes	Sindo
*Sordariales*	*Scytalidium thermophilum*	620.91	Saw dust&wood chips In pighouse bedding	Yes	Sther
*Onygenales*	*Malbranchea cinnamomea*	115.68	*Oryza sativa* (*Gramineae*) seeds	Yes	Mcinn
*Eurotiales*	*Talaromyces byssochlamydoides*	151.75	Desert soil	Poor^b^	Tbyss
*Eurotiales*	*Talaromyces emersonii*	393.64	Compost	Yes	Temer
*Eurotiales*	*Talaromyces leycettanus*	398.68	Coal spoil tip soil	+ YE^c^	Tleyc
*Eurotiales*	*Talaromyces thermophilus*	236.58	Decaying *Parthenium argentatum*	Yes	Tther
*Eurotiales*	*Thermoascus aurantiacus*	891.70	Wood	+ YE^c^	Taura
*Eurotiales*	*Thermomyces lanuginosus*	632.91	Rotting guayule shrub	Yes	Tlanu
*Mucorales*	*Rhizomucor miehei*	182.67	Retting *Parthenium argentatum*	Yes	Rmie
*Mucorales*	*Thermomucor indicae-seudaticae*	104.75	Municipal compost	Yes	Tindi

^a^Strain registration number at Centraalbureau voor Schimmelcultures.

^b^Good growth on avicel with 0.5% yeast extract.

^c^Only growth on avicel with 0.5% yeast extract.

For DNA purification the fungi were grown on 6% wheat bran (Finax, Esbjerg, Denmark), 1.5 % agar (Sigma-Aldrich, Cambridge, UK) plates at the recommended temperature.

### Endoglucanase assay

Endoglucanase activity was measured with Azo-CM-Cellulose (cat. S-ACMC, Megazyme, Bray, Ireland) as substrate in an assay modified from the manufacturer’s protocol. A culture broth sample of 20 μl was mixed with 20 μl of 2% Azo-CM-Cellulose in 2× McIlvaine Buffer (McIlvaine 1921) at the desired pH. The culture broth sample was incubated at the indicated time and temperature before mixing with 100 μl of precipitant (300 mM Na acetate, 18 mM Zn acetate, 76% EtOH (pH 5)). The stopped reactions were centrifuged 16,000 g for 1 minute and 100 μl of the supernatant was dried down at 80°C and resuspended in 5 μl of water. Finally, the absorption at 600 nm was measured and enzyme activity was calculated as described (Megazyme, Bray, Ireland).

### Filter paper assay

Degradation of filter paper was measured by first cutting a 5 × 5 mm piece of filter paper #1 (Whatman, Kent, UK) with a pair of sharp scissors whereafter it was submerged in 80 μl of 1.25x McIlvaine Buffer (McIlvaine 1921) at the desired pH. Next, 20 μl of sample was added and the assay was incubated at the indicated time and temperature. The filter paper was removed and 0.3 μl of Novozym 188 (Novozymes, Bagsvaerd, Denmark) was added and the sample incubated for 30 minutes at 50°C to convert any cellobiose to glucose. The reactions were centrifuged 16,000 g for 1 minute and the supernatant was dried down at 80°C and resuspended in 5 μl of water. Finally, the glucose content of the sample was measured with the D-Glucose HK Assay Kit (Megazyme, Bray, Ireland).

### DNA purification

Fungal mycelium was scraped of the top of a wheat bran agar plate, frozen in N_2_(l) and ground with a mortar and pestle. DNA was extracted from the homogenized mycelium with the Fungal DNA Mini Kit (Omega Bio-Tek, Norcross, GA, USA) according to the manufacturer's instructions.

### RNA purification

Fungal mycelium was collected by filtration of liquid cultures through Miracloth (Calbiochem, San Diego, CA, USA) and RNA was purified with a Total RNA kit (A&A Biotechnology, Gdynia, Poland) or with Tri Reagent (Sigma-Aldrich, St. Louis, MO, USA).

### Design of degenerated primers

For each of the glycoside hydrolase families 6 (GH6), 7 (GH7) and 45 (GH45) the most conserved hexapeptides were found in thermophilic and thermotolerant fungal enzymes available in GenBank. These hexapeptides were reverse translated according to the genetic code. Positions containing any nucleotide (A, C, G or T) were substituted with inosine (Table 2). Degenerate nucleotides at the 3' end of the primers were removed from the sequence of the primers. Reverse primers were designed to be complementary to the DNA sequence encoding the hexapeptide.

**Table 2:** Sequence of conserved peptides and PCR primers

**Target**	**Peptide**	**Primer sequence**^**a**^
GH6	LPDRDC	CAGGTCCTICCIGAYMGIGAYTG
GH6	GWLGWP	CAGGTCGGITGGCTIGGITGGC
GH6	GLATNV	CAGGTCGGICTIGCIACIAAYGT
GH6	PAPEAG	CAGGTCCCIGCYTCIGGIGCIGG
GH6	WFQAYF	CAGGTCAARTAIGCYTGRAACCA
GH6	WVKPGG	CTGGACCCICCIGGYTTIACCCA
GH6	GLATNV	CAGGTCGGICTIGCIACIAAYGT
GH6	VVYDLP	CTGGACGTIGTITAYGAYCTICC
GH7	DANWRW	CTGGACGAYGCIAAYTGGMGITGG
GH7	EFTFDVD	CTGGACGARTTYACITTYGAYGTIGA
GH7	GTGYCD	CTGGACGGIACIGGITAYTGYGA
GH7	EMDIWEA	CTGGACGCYTCCCADATRTCCATYTC
GH7	DGCDFN	CTGGACTTRAARTCRCAICCRTC
GH7	VVTQF	CTGGACAAYTGIGTIACIAC
GH45	YWDCCK	CAGGTCTAYTGGGAYTGYTGYAA
GH45	PGGGVG	CAGGTCCCIACICCICCICCIGG
GH45	WR(F/Y)(D/N)WF	CAGGTCAACCARTTRTAICKCCA
GH45	WCCACY	CTGGACTARCAIGCRCARCACCA
GH45	WCCACY	CTGGACTGGTGYTGYGCITGYTA
GH45	WDCCKP	CTGGACTGGGAYTGYTGYAARCC

^a^I = Inosine, Y = C and T, K = G and T, M = A and C, R = A and G.

A tail of six bases (CTGGAC) was added to the 5' end of all primer sequences as this improves the performance of short primers (Andersen et al. 2000; Balcells et al. 2011; Chen et al. 2005).

The primers were synthesized and HPLC-purified by Sigma-Aldrich (Cambridge, UK).

### PCR with degenerated primers

A mix of 100 ng total fungal DNA in 1x Run PCR buffer, 2 mM each dATP, dCTP, dGTP and dTTP, 400 nM forward primer; 400 nM reverse primer; 1U RUN DNA polymerase (A&A Biotechnology, Gdynia, Poland) in a total volume of 20 μl was used for PCR on an MyCycler (Bio-Rad, Hercules, CA, USA) with the following thermal profile:

Initial denaturation 95°C, 5 minutes.

30–40 cycles of 95°C, 20 seconds; annealing temperature, 30 seconds; 72°C, 60 seconds and a final extension at 72°C 5 minutes.

The number of cycles and the annealing temperature was optimized for each primer set (Table 2).

PCR products were analyzed by agarose gel electrophoresis and selected DNA were cut out and purified with the Qiaquick kit (Qiagen, Hilden, Germany).

One μl of the purified PCR product was reamplified in a 50 μl reaction under the same conditions as the original PCR except that only 15 to 20 cycles of PCR were performed.

### RT-PCR and RACE

FirstChoice® RLM-RACE Kit (Invitrogen, Grand Island, NY, USA) was used for cDNA synthesis and RACE according to the manufacturer’s protocol.

For RT-PCR 1 μl of the RACE cDNA reaction was used for PCR in a final volume of 20 μl containing 1x Run PCR buffer, 2 mM each dATP, dCTP, dGTP and dTTP, 500 nM forward primer; 500 nM reverse primer; 1U RUN DNA polymerase (A&A Biotechnology, Gdynia, Poland) on an MyCycler (Bio-Rad, Hercules, CA, USA) with the following thermal profile:

Initial denaturation 95°C, 5 minutes.

30 cycles of 95°C, 30 seconds; 60°C, 30 seconds; 72°C, 60 seconds and a final extension at 72°C 5 minutes.

PCR products were analyzed by agarose gel electrophoresis.

### Sequencing and analysis

PCR products were cycle sequenced by Eurofins-MWG (Ebersberg, Germany) or StarSEQ (Mainz, Germany) with one of the degenerated primers used for PCR.

The resulting sequences were translated to amino acid sequence and used for BLAST search (Altschul et al. 1997) against the non-redundant protein sequence database at NCBI and inspected for conserved domains (Marchler-Bauer et al. 2011) in the CDD database at NCBI.

Sequence alignment was made with ClustalW (Larkin et al. 2007) and adjusted manually. Phylogenetic trees were made with MUSCLE, PhyML and TreeDyn at Phylogeny.fr (Dereeper et al. 2008).

### Statistical analysis

P-values were calculated by two-sided Student’s T-test or by analysis of variance tests (ANOVA) unless indicated otherwise.

### Nucleotide sequence accession numbers

The DNA sequences of the product of PCR amplification have been deposited in the European Nucleotide Archive databases [EMBL:HG313865, HG313866, HG313868, HG313869, HG313871 - HG313874, HG313876 - HG313881 and HG313883 - HG313889].

## Results

To characterize and compare the cellulolytic potential of thermophilic fungi we chose one isolate of each of 16 (according to the provider) non-pathogenic, non-toxin-producing thermophilic fungi.

A medium with microcrystalline cellulose as the only carbon source was inoculated with each of the 16 fungal strains to test their ability to grow on cellulose. All of the fungi could grow on the microcrystalline cellulose. However, *Talaromyces leycettanus* and *T. aurantiacus* needed a supplement of 0.5% yeast extract for growth (Table 1). Also *Chaetomium senegalense* and *Talaromyces byssochlamydoides* grew better when the cellulose minimal medium was supplemented with yeast extract.

**Figure 1:**
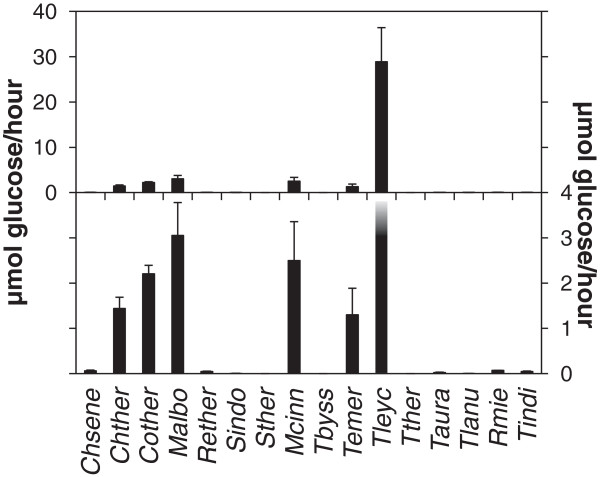
**Secreted endoglucanase activity from 16 thermophilic fungi grown on microcrystalline cellulose.** Endoglucanase activity of secreted fungal enzymes was measured as described in “Methods”. The left y-axis indicates the scale for the upper panel including endoglucanase activities from all the fungi and the right y-axis indicates the scale for the lower panel. The endoglucanase activity from *T. leycettanus* is truncated. Error bars indicate standard deviations. See Table 1 for abbreviations of fungal names. The figure represents the result of triple determination of the enzyme activity for each culture in one growth experiment. The growth experiment was repeated twice with similar results.

To assess the cellulolytic potential of the fungi when grown on microcrystalline cellulose samples of culture medium were removed at various times and assayed for endoglucanase activity. All of the fungi produced measurable levels of endoglucanase activity except *S. thermophilum, T. byssochlamydoides* and *Talaromyces thermophilus* where no endoglucanase activity was detectable in the medium (Figure 1) although these fungi grew well on cellulose (Table 1).

Of the 13 fungi where endoglucanase activity was detected the activity measured in the medium from *T. leycettanus* was more than five times higher than from *M. albomyces* (*P* < 0.001, Student’s T-test), which had the second highest endoglucanase activity of the fungi used in the present study (Figure 1).

**Figure 2:**
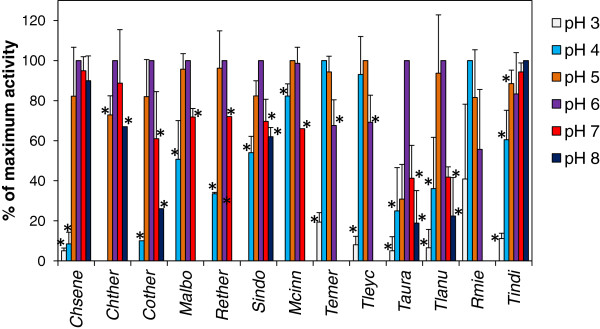
**Optimal pH for the secreted endoglucanase activities.** The culture supernatants from fungi grown on microcrystalline cellulose were brought to the pH indicated and the endoglucanase activity was measured. Activities are given as percent of the maximal measured endoglucanase activity. The experiment was repeated three times. The activities were determined as described in “Methods”. Error bars indicate standard deviations. See Table 1 for abbreviations of fungal names. Stars next to bars indicates that the activity at this pH was significantly lower than the highest activity (*P* < 0.05, Student’s T-test).

The pH optimum of the endoglucanase activity of each fungus was determined by performing the assay at different pH values (Figure 2). Most of the fungal endoglucanases had optimal activity at pH 4 – 6 but the highest activity of the endoglucanase from *Thermomucor indicae-seudaticae* was measured at pH 8.

The relatively broad pH optima measured for fungi like *C. senegalense* and *T. indicae-seudaticae* indicate that these fungi are able to degrade cellulose in environments with different pH values (Figure 2). The broad pH optimum may be due to one enzyme, which is active at several pH values, or to different enzymes with different pH optima. The only fungus among the species studied with highest activity at high pH value was the zygomycete *T. indicae-seudaticae*.

**Figure 3:**
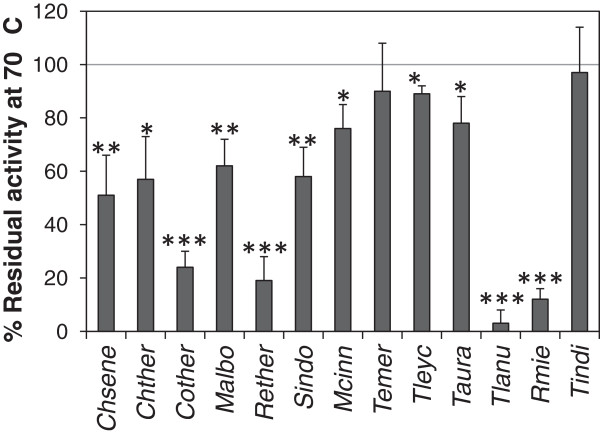
**Heat stability of the secreted endoglucanase activities.** Aliquots of the culture supernatants from fungi grown on microcrystalline cellulose were incubated for one hour at 70°C before measuring the endoglucanase activity (see “Methods”). Activities are given as percent remaining activity after one hour incubation at 70°C compared to control samples incubated at 50°C. The experiment was repeated three times. Error bars indicate standard deviations. Stars next to bars indicate that the activity at this pH was significantly lower than the highest activity (1 star: *P* < 0.05, 2 stars: *P* < 0.01, 3 stars: *P* < 0.001, Student’s T-test). See Table 1 for abbreviations of fungal names.

To assess thermostability of the endoglucanase activity the fungal extracts were incubated at 70°C for one hour before activity measurement. The activity after incubation at 50°C for one hour was used as reference. Thermostability varied from practically 100% activity after incubation at 70°C for one hour for the zygomycete *T. indicae-seudaticae* to almost complete loss of activity for the ascomycete *Thermomyces lanuginosus* (*Eurotiales*, Figure 3).

To access the total cellulolytic potential of the secretome from the 13 fungi with endoglucanase activity we measured the activity of the culture supernatants in a filter paper degradation assay. Degradation of filter paper requires the combined activity of both endo- and exocellulases and is often used as a measure of total cellulolytic potential (Dashtban et al. 2010; Zhang et al. 2009).

**Figure 4:**
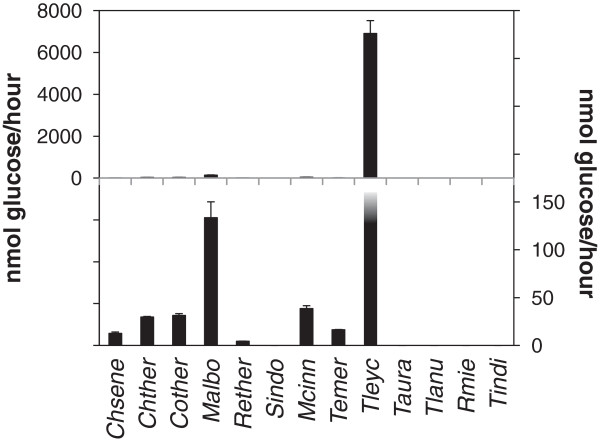
**Cellulolytic potential of thermophilic fungi grown on microcrystalline cellulose.** Cellulolytic potential was measured as filter paper degrading activity of secreted fungal enzymes as described in “Methods” for the fungi that had endoglucanase activity. The left y-axis indicates the scale for the upper panel including filter paper degrading activities from all the fungi that and the right y-axis indicates the scale for the lower panel. The filter paper degrading activity from *T. leycettanus* is truncated. The figure represents the result of triple determination of the enzyme activity for each culture in one growth experiment. Symbols as in Figure 1. See Table 1 for abbreviations of fungal names.

**Figure 5:**
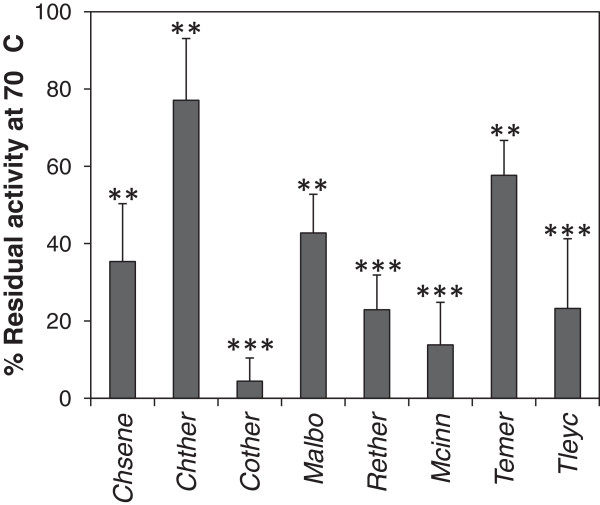
**Heat stability of the secreted cellulolytic enzymes.** Aliquots of the culture supernatants from fungi grown on microcrystalline cellulose were incubated for one hour at 70°C before measuring the filter paper degrading activity (see “Methods”). Activities are given as percent remaining activity after one hour incubation at 70°C percent compared to control samples incubated at 50°C. The experiment was repeated three times. Error bars indicate standard deviations. Stars next to bars indicate that the activity at this pH was significantly lower than the highest activity (1 star: *P* < 0.05, 2 stars: *P* < 0.01, 3 stars: *P* < 0.001, Student’s T-test). See Table 1 for abbreviations of fungal names.

The *T. leycettanus* secretome showed more than 50 times higher cellulolytic activity as measured in the filter paper assay than the second most active secretome (Figure 4, *P* = 0.003, Student’s T-test). Although only 23% of the activity remained after incubation at 70°C for one hour this is still much higher cellulolytic potential than the initial cellulolytic potential of any of the other fungi (Figure 5, *P* = 0.03, Student’s T-test)).

The loss of cellulolytic potential of *T. leycettanus* at 70°C does not correlate with the high thermostability of the endoglucanase activity of this fungus. This suggests that one or more of the cellobiohydrolases, monooxygenases or auxiliary enzymes that are involved in filter paper degradation are heat labile.

For *T. leycettanus* and the 12 other fungi where the endoglucanase activity was measurable this activity was either higher than the filter paper degrading activity or the filter paper degrading was too low to detect (Figures 2 and 4, *P* < 0.001, Student’s T-test). This result suggests that the release of cellulose polymers from an insoluble substrate is rate limiting compared to the decomposition of the released cellulose into oligomers. However, the data should be interpreted with care as it is difficult to compare activities measured in different assays towards different substrates.

To determine if the fungi have genes encoding endoglucanases and cellobiohydrolases we designed degenerated primers for PCR amplification of the GH45 endoglucanases and the GH6 and GH7 cellobiohydrolases. With these primers it was possible to amplify and sequence partial genes for six GH6, eight GH7 and eight GH45 from the fungi. All sequences were submitted to the European Nucleotide Archive database except for the GH7 from *Rhizomucor miehei* that can be found in Additional file 1. Expression of the genes was confirmed by RT-PCR of RNA isolated from fungi cultivated on microcrystalline cellulose medium (data not shown).

Together with the genes sequenced by others this provides positive confirmation that GH6, GH7 and GH45 are found in all three thermophilic orders of the *Ascomycota* and GH7 and GH45 are present in the *Mucorales* (Table 3). No GH6 were found in the two fungi of the order *Mucorales* but it cannot be excluded that *T. indicae-seudaticae* and *R. miehei* have genes encoding GH6 enzymes but that these genes were not amplified by the primers used in the present study.

**Table 3:** Genes encoding GH6, GH7 and GH45 enzymes sequenced in this study or found in GenBank

**Fungus**	**GH6**	**GH7**	**GH45**
*C. senegalense*	HG313865^a^	HG313873^a^	HG313881^a^
*C. thermophilum*	AAY88915.1^b^	AAW64926.1^b^	EGS20050.1^b^
*C. thermophilus*	HG313866^a^	HG313874^a^	HG313883^a^
*M. albomyces*	CAH05671.1^b^	CAD56667.1^b^	CAD56665.1^b^
*R. thermophila*	HG313871^a^	HG313878^a^	HG313886^a^
*S. indonesiacum*	HG313872^a^	HG313879^a^	HG313887^a^
*S. thermophilum*	BAB39154.1^b^	BAA09785.1^b^	BAA74956.1^b^
*M. cinnamomea*	CAH05679.1^b^	HG313889^a^	HG313885^a^
*T. byssochlamydoides*	HG313868^a^	HG313876^a^	NR^c^
*T. emersonii*	AAL33604.4^b^	AAL33603.2^b^	CAJ75963.1^b^
*T. leycettanus*	HG313869^a^	HG313877^a^	NR^c^
*T. thermophilus*	NR^c^	NR^c^	NR^c^
*T. aurantiacus*	NR^c^	CAM98447.1^b^	HG313884^a^
*T. lanuginosus*	NR^c^	ABR57319.1^b^	NR^c^
*R. miehei*	NR^c^	This study^a,d^	HG313888^a^
*T. indicae-seudaticae*	NR^c^	NR^c^	HG313880^a^

^a^This study.

^b^GenBank.

^c^NR: Not reported.

^d^Too short sequence for submission to the public databases but can be found as Additional file 1.

**Figure 6:**
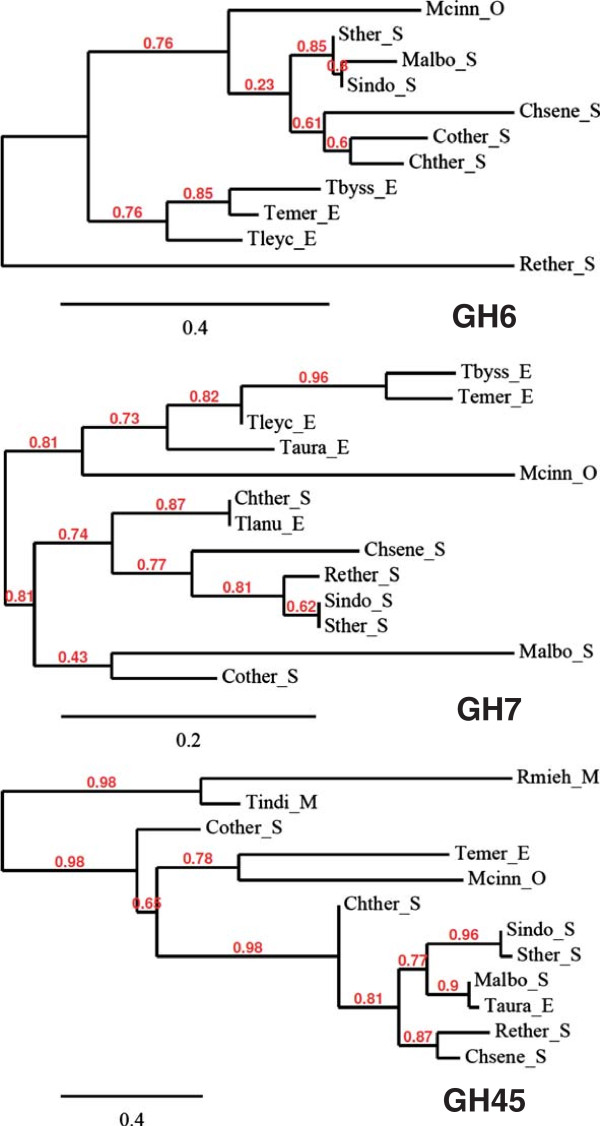
**Phylogeny of sequenced genes encoding GH6, GH7 and GH45 family proteins from the thermophilic fungi.** All the genes sequenced in the present study except GH7 from *R. miehei* that was too short to produce any significant alignment and the genes found in NCBI (see Table 3) were aligned with MUSCLE before curating the alignments with Gblocks and building of phylogenetic trees with PhyML (Dereeper et al. 2008). The three were depicted with TreeDyn. O: *Onygenales*; S: *Sordariales*; E: *Eurotiales*; M: *Mucorales*. See Table 1 for abbreviations of fungal names.

Phylogenetic analysis did not reveal any correlation between the thermostability and activity of the enzyme activity and the protein sequences (Figure 6).

## Discussion

In the present study we have assessed the cellulolytic potential of isolates of 16 different thermophilic fungi belonging to four orders by checking their growth on microcrystalline cellulose and characterizing their secreted cellulose-degrading enzymatic activities.

All of the fungal strains could grow on microcrystalline cellulose and were therefore able to degrade cellulose. Two of the fungi (*T. leycettanus* and *T. aurantiacus*) needed a supplement of yeast extract for growth. Two observations point to that the growth of these two fungi on cellulose was not limited by access to carbon but rather by some other factor that could be provided by the yeast extract. Firstly, yeast extract contains 0.4 g carbon per g (Holwerda et al. 2012). Therefore, the total contribution of non-cellulose carbon from the yeast extract is 0.2%, which will only allow limited growth of the fungi. Secondly, *T. aurantiacus* only grew poorly and *T. leycettanus* not at all on cellulose medium when the medium was supplemented with easily fermentable carbon in the form of 1% glucose (data not shown). Therefore it was not the amount of available carbon per se that was growth limiting. In support of this notion, *T. leycettanus* was very capable of degrading cellulose as the enzyme assays showed that it secreted by far the highest amount of cellulases of all the fungi tested.

Interestingly, the *M. albomyces* strain, which was isolated from chicken nest straw in Nevada, grew readily on the cellulose medium in contrast to what was previously reported for a strain of *M. albomyces* isolated from forest soil and compost in India (Maheshwari and Kamalam 1985). This suggests that different isolates of *M. albomyces* have different cellulolytic potential. Moreover, the *M. albomyces* strain used in the present study was the fungus with the second highest cellulolytic potential thereby supporting the notion that this strain is able to degrade cellulose in contrast to the previously characterized strain (Maheshwari and Kamalam 1985).

This difference suggests that it is difficult to conclude about growth preferences of a fungal species based on the study of a single strain. It is tempting to speculate that the original biotope of the strain used in the present study had higher cellulose content than the biotope of the strain used by Maheshwari and Kamalam (Maheshwari and Kamalam 1985).

Likewise, *T. lanuginosus* is generally considered to be unable to utilize cellulose as sole carbon source (Maheshwari et al. 2000) in contrast to the observation in the present study. However, different strains of *T. lanuginosus* have different ability to grow on cellulose (Markowska-Szczupak et al. 2012) in agreement with that there can be large differences in the cellulolytic potential of different isolates of the same species.

This notion is supported by the absence of detectable endoglucanase activity from the strain of *S. thermophilum* used in the present study whereas it has been reported that other strains of *S. thermophilum* produce endoglucanase and other cellulases when grown on recalcitrant polysaccharides (Hayashida and Mo 1986; Ögel et al. 2001; Poças-Fonseca et al. 2000).

It is difficult to account for that some of the fungi e.g. *S. thermophilum* could grow on cellulose without producing any detectable endoglucanase activity. It cannot be completely ruled out that the cellulose-degrading activity is membrane associated but it is highly unlikely as no endoglucanase activity was detected in the supernatant from homogenized mycelium (data not shown). Furthermore, it is quite unusual for fungal cellulases to be membrane-anchored although it has been reported. One example is a membrane-anchored endoglucanase that was cloned from *Phanerochaete chrysosporium* (Vanden Wymelenberg et al. 2002). Another possibility is that *S. thermophilum* uses a different mechanism such as oxidative processes to degrade the cellulose (Arantes et al. 2011; Rineau et al. 2012). However, this fungus can express cellulose-degrading activity (Ögel et al. 2001) and has genes encoding endoglucanases (Takashima et al. 1999).

One purpose of the present study was to elucidate whether it is possible to predict high thermostability from fungal taxonomy. However, there was no obvious correlation between the fungal orders and the thermostability but at genus level the enzymes from the two species of *Talaromyces* with measurable endoglucanase activity showed high thermostability. As the cellulolytic potential of some of the isolates used in the present study was different from the cellulolytic potential reported for other isolates of the same species (Hayashida and Mo 1986; Maheshwari et al. 2000; Maheshwari and Kamalam 1985; Markowska-Szczupak et al. 2012; Ögel et al. 2001; Poças-Fonseca et al. 2000) it is likely that the thermostability of enzymes may vary between isolates of the same species. However, the high thermostability observed for the enzyme activity from *T. emersonii* and *T. aurantiacus* (both *Eurotiales*) is in agreement with previous reports on these fungi (Gomes et al. 2000; Hong et al. 2003; Khandke et al. 1989; McClendon et al. 2012; Murray et al. 2004; Parry et al. 2002; Romanelli et al. 1975; Voutilainen et al. 2010).

Just like the taxonomy, the predicted protein sequences of the sequenced genes did not exhibit any correlation to the thermostability of the cellulose-degrading activity. Instead the protein sequences grouped largely according to the taxonomic order with a few exceptions. E.g., the GH45 sequences from *Corynascus thermophilus* did not fall in the same branch of the phylogenetic tree as the other GH45 from *Sordariales*. Moreover, the GH45 protein from *T. aurantiacus* shows higher relationship to the protein from *M. albomyces* from *Sordariales* than to the protein for the other fungus of the *Eurotiales* order, *T. emersonii*. Similar results were found for the DNA sequences of the enzyme-encoding genes (Additional file 2).

One curious result was that no GH6 encoding genes were found in the two fungi of the order *Mucorales*. Although it is possible that these fungi have some GH6 genes that were simply not found in the present study, it is interesting that another Z*ygomycota* of the *Mucorales* order, *Rhizopus oryzae*, is able to degrade cellulose despite the absence of any GH6 gene in its genome (Battaglia et al. 2011).

The high pH optimum of the cellulolytic activity from *T. indicae-seudaticae* distinguished this fungus from the other fungi. Also a purified glucoamylase from *T. indicae-seudaticae* has a relatively high pH optimum (Kumar and Satyanarayana 2003) suggesting that this fungus may be adapted to growth in an environment with high pH.

For six of the eight fungi where high activity was measured in both assays the endoglucanase activity was more heat stable than the filter paper degrading activity (Figure 3 and 5, *P* = 0.012, Student’s T-test). This is in agreement with that more enzymes including endoglucanases are necessary for filter paper degradation than for decomposition of carboxy-methyl cellulose (reviewed by (Dashtban et al. 2010)). In view of this it seems surprising that *C. thermophilum* exhibited more stable filter paper degrading activity than endoglucanase activity (Figures 3 and 5). This can be explained by the finding that the endoglucanase activity of *C. thermophilum* measured as μmoles of glucose released per hour was almost 70 times higher than the filter paper degrading activity. Therefore the endoglucanase activity was not rate limiting for the total cellulolytic potential of the fungus.

In conclusion, the results of the present study show that thermophilic fungi of all four orders from where thermophilic species have been described, are able to degrade cellulose. The main differences in cellulolytic potential and thermostability of the secretome do not seem to correlate to the fungal order.

## Competing interests

Both authors are designated as inventors of a patent on the cloned genes originally filed by Aalborg University and later transferred to Novozymes A/S. Neither of the authors have any personal financial interests in the patent but part of the authors' research is funded through a grant from Novozymes A/S.

## Supplementary Material

Additional file 1 **Sequence of the GH7 gene fragment from *R. miehei.*** DNA sequence of the truncated GH7 gene that was amplified and sequenced from *R. miehei*. The lower sequence is the amino acid sequence of the fragment translated in the frame that resembles a GH7 family protein. Click here for file

Additional file 2 Phylogenetic relationship of the DNA sequences encoding enzymes belonging to the GH6, GH7 and GH45 families from the thermophilic fungi. Click here for file
