# Supplementary material for: Cellulolytic potential of thermophilic species from four fungal orders
Source: AMB Express. 2013 Aug 19;3:47. doi: 10.1186/2191-0855-3-47 (PMC3766086; doi:10.1186/2191-0855-3-47)
Supplement: Additional file 1 — Sequence of the GH7 gene fragment from R. miehei. DNA sequence of the truncated GH7 gene that was amplified and sequenced from R. miehei. The lower sequence is the amino acid sequence of the fragment translated in the frame that resembles a GH7 family protein. [file 2191-0855-3-47-S1.pdf]

Additional file 1

Cellulolytic potential of thermophilic species from four fungal orders.

AMB Express

Peter Kamp Busk and Lene Lange
Department of Biotechnology Chemistry and Environmental Engineering
A.C. Meyers Vænge 15, 2450 Copenhagen SV, Denmark. pkb@bio.aau.dk.

>Rhizomucor miehei GH7 DNA

ggcatcggcaacatcgagggcaagggctcgtgctgcaacgagatggatatttgggaggcg

>Rhizomucor miehei GH7 translated

GIGNIEGKGSCCNEMDIWEA

Additional file 1: Sequence of the GH7 gene fragment from *Rh. miehei*.
DNA sequence of the truncated GH7 gene that was amplified and sequenced from *Rh. miehei*. The lower sequence is the amino acid sequence of the fragment translated in the frame that resembles a GH7 family protein.
