# Supplementary material for: Cellulolytic potential of thermophilic species from four fungal orders
Source: AMB Express. 2013 Aug 19;3:47. doi: 10.1186/2191-0855-3-47 (PMC3766086; doi:10.1186/2191-0855-3-47)
Supplement: Additional file 2 — Phylogenetic relationship of the DNA sequences encoding enzymes belonging to the GH6, GH7 and GH45 families from the thermophilic fungi. [file 2191-0855-3-47-S2.pdf]

Additional file 2

Cellulolytic potential of thermophilic species from four fungal orders.

AMB Express

Peter Kamp Busk and Lene Lange
Department of Biotechnology Chemistry and Environmental Engineering
A.C. Meyers Vænge 15, 2450 Copenhagen SV, Denmark. pkb@bio.aau.dk.

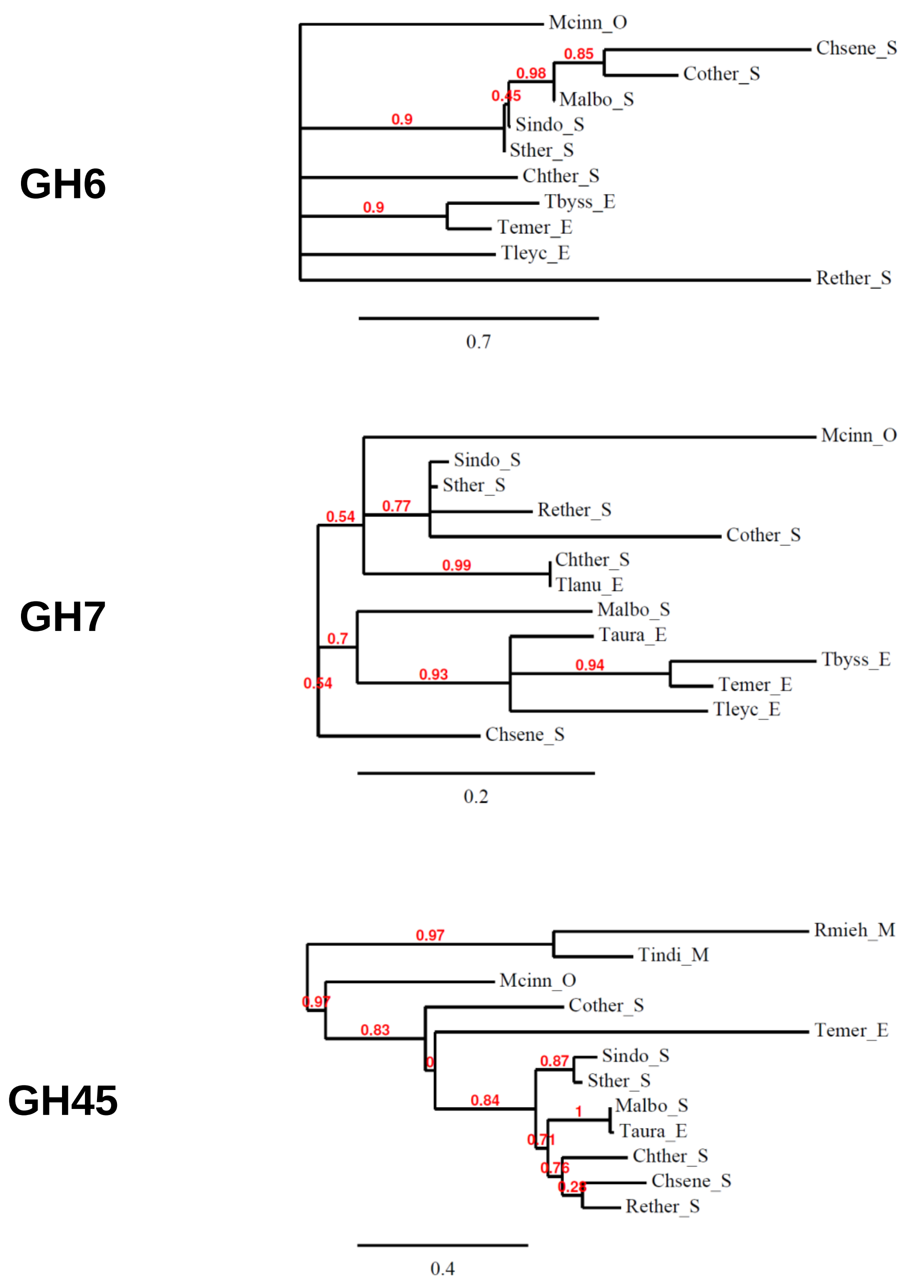

Additional file 2: Phylogenetic relationship of the DNA sequences encoding enzymes belonging to the GH6, GH7 and GH45 families from the thermophilic fungi.

All the genes sequenced in the present study except GH7 from R. miehei that was too short to produce any significant alignment and the genes found in NCBI (see Table 3) were aligned with MUSCLE before curating the alignments with Gblocks and building of phylogenetic trees with PhyML (Dereeper et al. 2008). The three were depicted with TreeDyn.

O: *Onygenales*; S: *Sordariales*; E: *Eurotiales*; M: *Mucorales*.

Chsene: *C. senegalense*; Chther: *C. thermophilum*; Cother: *C. thermophilus*; Malbo: *M. albomyces*; Rether: *R. thermophila*; Sindo: *S. indonesiacum*; Sther: *S. thermophilum*; Mcinn: *M. cinnamomea*; Tbyss: *T. byssochlamydoides*; Temer: *T. emersonii*; Tleyc: *T. leycettanus*; Tther: *T. thermophilus*; Taura: *T. aurantiacus*; Tlanu: *T. lanuginosus*; Rmie: *R. miehei*; Tindi: *T. indicae-seudaticae*.
